# Neuropsychological Profile of College Students Who Engage in Binge Drinking

**DOI:** 10.3389/fpsyg.2022.873654

**Published:** 2022-04-15

**Authors:** Jae-Gu Kang, Myung-Sun Kim

**Affiliations:** Department of Psychology, Sungshin Women’s University, Seoul, South Korea

**Keywords:** binge drinking, California Verbal Learning Test, executive function, non-verbal memory, Rey-Osterrieth Complex Figure Test, verbal memory, Wisconsin Card Sorting Test

## Abstract

This study investigated the neuropsychological profile of college students who engage in binge drinking (BD) using comprehensive neuropsychological tests evaluating verbal/non-verbal memory, executive functions, and attention. Groups were determined based on scores on the Korean version of the Alcohol Use Disorder Identification Test (AUDIT-K) and Alcohol Use Questionnaire (AUQ). There were 79 and 81 participants in the BD and non-BD groups, respectively. We administered the Korean version of the California Verbal Learning Test (K-CVLT) and Rey-Osterrieth Complex Figure Test (RCFT) to evaluate verbal and non-verbal memory, respectively, and measured executive functions using the Wisconsin Card Sorting Test (WCST), Trail-Making Test, Controlled Oral Word Association Test and Stroop Color-Word Test. We administered the d2 test to evaluate attention. Neuropsychological performance was analyzed by multivariate analysis of variance. The BD group showed significantly poorer performance in the long-term free recall condition of the K-CVLT and delayed recall condition of the RCFT and completed significantly fewer categories on the WCST than the non-BD group. In addition, there were significant negative associations among the AUDIT-K total score, AUQ binge score, and long-term free recall score of the K-CVLT. There were significant negative associations between the total AUDIT-K score and delayed recall RCFT score, and between the total AUDIT-K total score and numbers of completed categories on the WCST. These results indicate that college students who participate in BD have difficulties with verbal/non-verbal memory and executive functions, and further suggest that excessive alcohol use could have detrimental effects on the hippocampal-prefrontal circuit even with a relatively short period of alcohol use.

## Introduction

It has long been accepted that chronic alcohol use has deleterious effects on the brain, leading to alcohol-related brain damage (Harper, 2009; Fritz et al., 2019). Individuals with alcohol-related brain damage display significant impairments in memory (Race and Verfaellie, 2012) and executive function (Maharasingam et al., 2013), in which dysfunctional hippocampal-prefrontal circuitry is implicated (Nunes et al., 2019).

Neuroimaging studies have shown that excessive alcohol consumption during adolescence or early adulthood, when the prefrontal cortex and parietal and temporal regions are still developing, is more detrimental to the brain than alcohol consumption at later times (Arnett, 2005; Spear, 2013). Binge drinking (BD), a pattern of drinking a large amount of alcohol within a short period followed by a period of abstinence (Wechsler and Nelson, 2001; Maurage et al., 2013), has attracted growing interest because BD is most prevalent among young adults, especially college students (Chun et al., 2003; Stephens and Duka, 2008). Although some recent studies have attempted to conceptualize and redefine BD (Maurage et al., 2020; Lannoy et al., 2021), BD is usually defined based on the quantity, speed, and frequency of alcohol consumption, as follows: 5 and 4 units of alcohol in males and females, respectively, more than once during the past 2 weeks; or 5 and 4 units of alcohol over a 2-h period, in males and females, respectively, leading to a blood alcohol concentration of 0.08 g/dL (Wechsler and Nelson, 2001; National Institute of Alcohol Abuse and Alcoholism [NIAAA], 2004).

Studies using structural neuroimaging techniques have reported structural abnormalities of cortical and subcortical areas in adolescents and young adults with BD. Reduced gray matter volumes, and thinner cortical tissues in frontal and temporal areas were observed in adolescents with BD compared to a non-BD group. In the former group, there was also a negative correlation between the number of binging episodes and frontal/parietal cortical thickness (Pfefferbaum et al., 2016). Subcortical areas, including the hippocampus (Medina et al., 2007; Meda et al., 2018) and cerebellum (Lisdahl et al., 2013), also exhibited decreased volumes in adolescents and young adults with BD compared to those without BD.

Functional alterations of brain systems involved in several cognitive domains, including working memory, inhibition and learning/memory, were also found in adolescents and young adults who engaged in BD. For example, young adults with BD exhibited greater activity in the dorsomedial prefrontal cortex during a working memory task (Campanella et al., 2013), and adolescents with BD showed decreased activity in frontal regions during a spatial working memory task compared to those without BD (Squeglia et al., 2011). In addition, young adults with BD exhibited greater activity in the frontal cortex, anterior cingulate cortex and insular during the Go/NoGo task, which measures inhibition ability, than those without BD (Ames et al., 2014). During a verbal paired associates task, adolescents with BD exhibited decreased activity in the inferior frontal region, but increased activity in the dorsal frontal and parietal regions, compared to those without BD (Schweinsburg et al., 2011).

In line with neuroimaging results, adolescents and young adults with BD exhibit impairments in a variety of cognitive domains. Particularly, neuropsychological studies of BD have focused on executive functions and memory/learning, which are mainly controlled by the prefrontal cortex and medial temporal cortex, respectively. Among the executive functions, inhibition, decision-making, and working memory have been studied most extensively (Carbia et al., 2018). Young adults with BD showed poorer performance on inhibition tasks, which measure the ability to inhibit pre-potent responses or mental representations, than those without BD (Czapla et al., 2015), although other studies did not observe significant differences between them (Moreno et al., 2012; Salas-Gomez et al., 2016). In addition, young adults with BD showed impaired decision-making abilities, which were measured by the Iowa Gambling Task (Xiao et al., 2009; Yoo and Kim, 2016). Regarding working memory, particularly spatial working memory, young female adults with BD exhibited poor performance (Townshend and Duka, 2005; Scaife and Duka, 2009), although other studies did not find significant differences between BD and non-BD female groups (Hartley et al., 2004). Adolescents and young adults with BD also showed verbal memory dysfunction (Carbia et al., 2017; Meda et al., 2018), although other studies did not observe significant differences in performance on verbal memory tasks between young adult BD and non-BD groups (Salas-Gomez et al., 2016).

The contradictory findings of neuropsychological and functional neuroimaging studies seem to be related to the use of different paradigms, tasks and definitions of BD. For example, the Go/NoGo task (Ames et al., 2014), Stop Signal task (Fernie et al., 2010) and Stroop Color-Word task (Salas-Gomez et al., 2016) have all been used to evaluate inhibition ability. Also, although most studies agreed regarding the definition of BD, they used different criteria in terms of the frequency of binge episodes (Severine et al., 2019).

Given that BD in college increases the likelihood of future development of alcohol use disorder (AUD) (O’Neill et al., 2001; Jennison, 2004), and the fact that college students with BD have difficulties with academic and social adjustment (Cha, 2005; Haller et al., 2010), the present study investigated the neuropsychological profile of college students who engage in BD using comprehensive neuropsychological tests. Neuropsychological assessments in various cognitive domains would promote understanding of the nature and degree of cognitive impairment, as well as its functional implications for individuals with BD. A neuropsychological profile also provides valuable insight regarding the most appropriate prevention and intervention strategies for individuals who engage in BD.

## Materials and Methods

### Participants

The participant-selection procedures used herein have been described in previous studies by our research group (Yoo and Kim. 2016). We administered the Korean version of the Alcohol Use Disorder Identification Test (AUDIT-K, Barbor et al., 1992; Lee et al., 2000) and Alcohol Use Questionnaire (AUQ, Mehrabian and Russell, 1978), and a questionnaire containing items about the frequency of BD episodes in the previous 2 weeks and age of onset of alcohol consumption, to 1,030 college students.

Participants with total scores of 12–25 on the AUDIT-K (Kim et al., 1999; Lee et al., 2000) who drank five (male) or four (female) glasses more than once during the last 2 weeks (Wechsler and Nelson, 2001), and more than three (male) or two (female) glasses per hour (National Institute of Alcohol Abuse and Alcoholism [NIAAA], 2004), were included in the BD group. A score ≥26 on the AUDIT-K indicates the possibility of alcohol dependence (Kim et al., 1999). Those with total scores less than 8 on the AUDIT-K who drank less than five (male) or four (female) glasses during the last 2 weeks, and less than two (male) or one (female) glasses per hour, were included in the non-BD group. One glass contains approximately 12 g ethanol. Thus, in this study, BD was defined based on the quantity, frequency and speed of alcohol consumption.

The Korean version of the Children of Alcoholics Screening Test (CAST-K, Jones, 1983; Kim et al., 1995) was administered as parents’ alcohol use can affect their offspring’s alcohol use (Ary et al., 1993). A score ≥6 on the CAST-K indicates the possibility of participants’ parents had a history of AUD (Kim et al., 1995), therefore, participants who scored ≥6 score were excluded from this study. In addition, the Korean Wechsler Intelligence Scale (KWIS, Yum et al., 1992), Self-Rating Depression Scale (SDS, Zung et al., 1965), and State-Trait Anxiety Inventory (STAI, Spielberger et al., 1970) were administered to control for levels of intelligence, depression, and anxiety, respectively, since intelligence and emotion can affect performance of the neuropsychological tests (Lezak, 2012). To ensure that participants did not have neurological disorders, psychiatric disorders, or drug/alcohol abuse, the Structured Clinical Interview for DSM-IV-Non Patient (SCID-NP, First et al., 1996) was administered.

Following application of the inclusion and exclusion criteria, and the exclusion of those who refused to participate, 79 (males, *n* = 22; females, *n* = 57; age range, 18 ∼ 27 years) and 81 (males, *n* = 17; females, *n* = 64; age range, 18 ∼ 27 years) participants were classified into the BD and non-BD groups, respectively. This study was approved by Sungshin Women’s University Institutional Bioethics Review Board (SSWUIRB, 2019-020). All participants provided written informed consent after receiving a complete description of the study and received remuneration for their participation.

### Neuropsychological Measurements

For the evaluation of verbal and visuospatial memory, the Korean version of California Verbal Learning Test (K-CVLT) and Rey-Osterrieth Complex Figure Test (RCFT) were administered, respectively. The K-CVLT (Delis et al., 1987; Kim and Kang, 1999) consists of five free-recall trials of list A, followed by a free-recall trial of list B and short-term free and cued recalls of list A. After a 20-min delay, long-term free/cued recalls of list A and recognition tests were administered. The total numbers of responses to free recall trials 1–5, free recall of list B, and the short- and long-term free-recalls of list A, were recorded. The RCFT, which was administered to evaluate visuospatial memory (Lezak, 2012), involves three conditions: copying, immediate recall (3 min after copying), and 30-min delayed recall, and a recognition trial. Accuracy and response times were calculated for each condition based on the system developed by Meyers and Meyers (1995).

Executive functions were evaluated by the Wisconsin Card Sorting Test (WCST), Trail-Making Test (TMT), Controlled Oral Word Association Test (COWA), and Stroop Color Word Test. The WCST, which requires sorting cards based on color, number, and shape, measures problem-solving, abstract thinking, and mental-set shifting (Lezak, 2012). The numbers of total errors, perseverative errors, and categories completed were determined based on the scoring system developed by Heaton et al. (1993). The TMT consists of two parts, A and part B, which involve connecting digits with a line, and alternately connecting digits and letters, respectively. The TMT is sensitive for evaluating the ability to shift mental sets and control attention (Lezak, 2012). The total numbers of errors and response times were scored. The COWA, which is widely used to evaluate frontal lobe functions including controlled attention (Stuss and Benson, 1986), requires participants to respond to many words beginning with a particular letter and belonging to a particular category. The total numbers of responses were recorded by letter and category. The Stroop Color-Word Test is widely used for measuring interference control (Rapport et al., 2001), and the numbers of words read correctly during 45 s were counted (Golden, 1978). The interference control was calculated as follows; score of color-word condition–[(score of word condition × score of color condition) / (score of word condition + score of color condition)] (Golden, 1978; van Mourik et al., 2005).

The d2 test (Brickenkamp and Zillmer, 1998), which measures selective attention, requires participants to detect a target as quickly and accurately as possible. The total number of errors was determined, and concentration index was measured by subtracting number of commission errors from the total number of correct responses. Neuropsychological tests were administered during a single session that lasted about 2 h.

### Statistical Analysis

The demographic characteristics of the BD and non-BD groups were analyzed using independent *t*-tests. Neuropsychological performance was analyzed by multivariate analysis of variance (MANOVA). All *p*-values were Bonferroni-corrected, and *p* < 0.05 was considered to be statistically significant. Associations between performance on the neuropsychological tests and BD severity were analyzed using the Pearson product-moment correlation coefficient. All statistical analyses were carried out using SPSS software (version 26.0; IBM Corp., Armonk, NY, United States). The Kolmogorov-Smirnov test and the Q-Q plot were used to ascertain whether data was normally distributed (Vetter, 2017).

## Results

### Demographic Characteristics

The demographic characteristics of the BD and non-BD groups are described in Table 1. The two groups did not differ in age [*t*(158) = −0.30, *p* = 0.77], educational level [*t*(158) = −0.52, *p* = 0.60], SDS score [*t*(158) = 0.64, *p* = 0.52], STAI state anxiety score [*t*(158) = 0.64, *p* = 0.52], STAI trait anxiety score [*t*(158) = 1.63, *p* = 0.10], or total IQ score on the KWIS [*t*(158) = −0.76, *p* = 0.45]. However, the groups differed significantly in terms of the total AUDIT-K score [*t*(158) = 33.59, *p* < 0.001], drinking speed [*t*(158) = 21.00, *p* < 0.001], frequency of drunkenness within the last 6 months [*t*(158) = 7.31, *p* < 0.001], percentage of drinking occasions resulting in drunkenness [*t*(158) = 7.85, *p* < 0.001], and AUQ binge score [*t*(158) = 16.35, *p* < 0.001].

**TABLE 1:** Demographic characteristics of the binge drinking and non-binge drinking groups.

	Non-binge drinking group (*n* = 81)		Binge drinking group (*n* = 79)		*t*
	Mean (SD)		Mean (SD)		
Age (years)	21.72	(2.40)	21.61	(2.18)	−0.30
Education (years)	14.94	(1.25)	14.82	(1.54)	−0.52
SDS	40.96	(7.46)	41.68	(6.72)	0.64
STAI state	40.25	(10.70)	41.28	(9.63)	0.64
STAI trait	41.35	(9.74)	43.81	(9.33)	1.63
KWIS total IQ	114.37	(9.90)	113.28	(8.08)	−0.76
AUDIT-K	1.67	(1.90)	17.52	(3.79)	33.59***
Drinking speed (drinks/hour)	0.75	(0.56)	4.23	(1.38)	21.00***
Frequency of drunkenness within the last 6 months	0.12	(0.43)	7.14	(8.63)	7.31***
Percentage of drinking occasions resulting in drunkenness (%)	11.27	(25.19)	46.23	(30.91)	7.85***
AUQ binge drinking score	5.40	(5.65)	33.33	(14.27)	16.35***

***p < 0.001.

*SDS, self-rating depression scale; STAI, Spielberger’s state-trait anxiety inventory; KWIS, the Korean Wechsler intelligence scale; AUDIT-K, the Korean version of alcohol use disorder identification test; AUQ, alcohol use questionnaire.*

### Neuropsychological Measures

**FIGURE 1:**
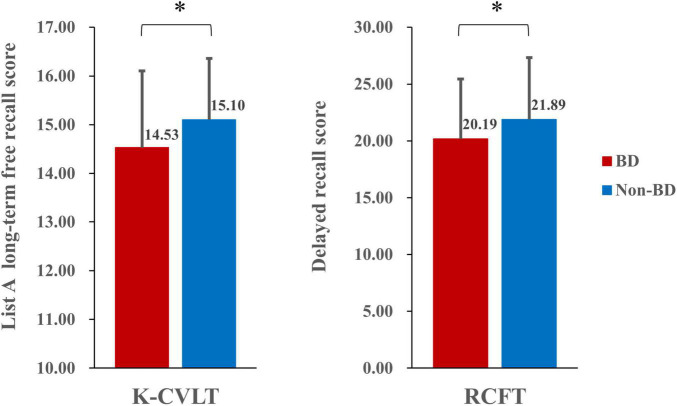
Performance of the Korean version of the California Verbal Learning Test (K-CVLT) and Rey-Osterrieth Complex Figure Test (RCFT) in the binge and non-binge drinking groups. *p < 0.05.

The MANOVA revealed significant differences in performances on the K-CVLT, RCFT, and WCST between the BD and non-BD groups. In terms of the K-CVLT, the BD group showed significantly poorer long-term free recall performance for list A than the non-BD group [*F*_(1,158)_ = 6.33, *p* = 0.013, η^2^_*p*_ = 0.039]. Individuals with BD also exhibited lower scores in the delayed recall condition of the RCFT [*F*_(1,158)_ = 4.03, *p* = 0.046, η^2^_*p*_ = 0.025] and completed fewer categories on the WCST [*F*_(1,158)_ = 3.97, *p* = 0.048, η^2^_*p*_ = 0.024] compared to those without BD. The performances of the BD and non-BD groups on the neuropsychological measures are presented in Table 2. Figure 1 presents the performances of the BD and non-BD groups on the long-term free recall of the K-CVLT and delayed recall condition of the RCFT.

**TABLE 2:** Neuropsychological performance of the binge drinking and non-binge drinking groups.

	Non-binge drinking group (*n* = 81)		Binge drinking group (*n* = 79)		*p*
	Mean (SD)		Mean (SD)		
RCFT					
*Response time (ms)*					
Copy	147.90	(41.56)	143.58	(52.82)	0.566
Immediate recall	176.48	(89.95)	159.03	(64.03)	0.160
Delayed recall	118.75	(49.72)	117.24	(48.34)	0.846
*Accuracy*					
Copy	33.05	(1.96)	32.31	(2.82)	0.056
Immediate recall	21.80	(5.40)	20.47	(5.46)	0.124
Delayed recall	21.89	(5.45)	20.19	(5.25)	0.046*
Recognition	19.99	(2.52)	20.35	(1.99)	0.309
K-CVLT					
List A trials 1–5	66.26	(7.91)	64.46	(8.11)	0.156
List B trial	9.09	(2.24)	8.85	(2.40)	0.517
List A short-term free recall	14.46	(1.57)	13.99	(2.10)	0.111
List A long-term free recall	15.10	(1.26)	14.53	(1.58)	0.013*
WCST					
Total number of errors	14.52	(9.46)	16.86	(14.04)	0.217
Perseverative errors	7.77	(5.07)	8.13	(6.69)	0.701
Categories completed	5.91	(0.50)	5.65	(1.10)	0.048*
TMT					
*Response time (ms)*					
Part A	27.93	(9.31)	26.52	(8.28)	0.314
Part B	57.80	(19.96)	55.54	(15.72)	0.428
*Error*					
Part A	0.01	(0.11)	0.00	(0.00)	0.325
Part B	0.21	(0.49)	0.22	(0.50)	0.946
COWA					
Letter	44.32	(11.10)	42.67	(8.52)	0.294
Category	39.33	(8.53)	38.71	(15.72)	0.642
Stroop					
*Accuracy*					
Word	84.90	(10.44)	85.50	(13.83)	0.758
Color	73.99	(10.11)	74.01	(11.21)	0.988
Word/Color	54.49	(11.76)	55.50	(9.82)	0.560
Interference Control	15.11	(10.61)	16.00	(7.43)	0.547
d2 Test					
Total number of errors	16.25	(15.16)	15.75	(12.93)	0.823
Concentration performances	227.06	(39.33)	228.00	(37.36)	0.877

*p < 0.05.

*RCFT, rey-osterieth complex figure test; K-CVLT, the Korean version of the California verbal learning test; WCST, Wisconsin card sorting test; TMT, trail-making test; COWA, controlled oral word association test; Stroop, stroop color-word test.*

### Correlations Between Binge Drinking and Performance on the Neuropsychological Tasks

There were significant negative correlations between the AUDIT-K total score and long-term free recall score for list A of the K-CVLT [*r* = −0.190, *p* < 0.01], and between the AUQ binge score and long-term free recall score for list A of the K-CVLT [*r* = −0.198, *p* < 0.01]. In addition, there were significant negative associations between the AUDIT-K total score and delayed-recall RCFT score [*r* = −0.161, *p* < 0.05], and between the AUDIT-K total score and numbers of completed categories of the WCST [*r* = −0.173, *p* < 0.05]. These correlations were observed in individuals with BD, but not in those without BD.

## Discussion

Since cognitive impairment affects the functional outcomes of patients with AUD (Heirene et al., 2016), and cognitive difficulties observed in patients with AUD are also found in individuals with BD (Mota et al., 2013), the present study investigated the neuropsychological profile of college students who engage in BD. The BD and non-BD groups exhibited significant differences in performance on the measures evaluating verbal/non-verbal memory and cognitive flexibility.

College students with BD performed worse in terms of long-term free recall of list A of the K-CVLT than those without BD. In addition, there were significant negative correlations of the AUDIT-K total score and AUQ binge score with the long-term free recall score for list A of the K-CVLT. In other words, individuals with BD have difficulties with verbal memory, where additional alcohol consumption correlated with poorer performance on a scale measuring long-term verbal memory.

Previous studies have also reported that young adults with BD have verbal memory difficulties. For example, Parada et al. (2011), Mota et al. (2013), and Carbia et al. (2017) found that college students with BD performed worse in the immediate and delayed recall conditions of the logical memory subtest of the Wechsler Memory Scale-III than those without BD. Sneider et al. (2013) also observed that young adults with BD recalled significantly fewer words on trials 1∼5 of the CVLT than those without BD. In addition, Meda et al. (2018) investigated the effect of alcohol consumption on the hippocampus and parahippocampus in college students over 2 years, and found that a higher alcohol use index, i.e., greater alcohol consumption, was associated with an accelerated decline of gray matter in the hippocampus/parahippocampus, and a larger reduction of hippocampal volume was in turn associated with poor memory performance, as measured by the CVLT. Therefore, the present results indicate that college students who participate in BD have difficulties in verbal memory, and these difficulties seem to be related to structural alterations of medial temporal regions including the hippocampus which are involved in verbal memory function.

Individuals with BD also showed poorer performance in the delayed recall condition of the RCFT compared to those without BD, and there was a significant negative association between the total AUDIT-K score and score in the delayed recall condition of the RCFT. These results are consistent with those of previous studies reporting poorer performance in the delayed recall condition of the RCFT in adolescents and young adults with BD compared to those without BD (Hartley et al., 2004; Squeglia et al., 2009; Winward et al., 2014; Kim et al., 2020).

Although the mechanisms underlying the visual memory difficulties observed in individuals with BD are not yet fully understood, alterations in functional connectivity of the brain seem to be related to visual memory impairments. For example, Kim et al. (2020) observed significantly lower delayed recall RCFT score in college students with BD compared to those without BD, and a significant positive association between left prefrontal-parietal occipital midline functional connectivity and performance in the delayed recall condition of the RCFT only in the BD group.

The RCFT is also known to be sensitive for assessment of executive functions, including organization and planning. For example, previous studies found that organizational strategies, evaluated *via* qualitative analysis of RCFT data, mediated visual memory deficits in patients with obsessive-compulsive disorder (Savage et al., 1999; Shin et al., 2004) and schizophrenia (Kim et al., 2008). Although studies about associations between executive functions and non-verbal memory using the RCFT in individuals with BD have not yet been reported, the difficulties with non-verbal memory observed in the present study could reflect difficulties of executive functions, such as organization and planning, in college students with BD.

The WCST, which is sensitive to prefrontal dysfunction (Demakis, 2003), has been widely used to evaluate executive functions (Rabin et al., 2005). Successful performance of the WCST requires strategic planning, the ability to use environmental feedback to shift cognitive set, goal-directed behavior, and the ability to inhibit impulsive responding (Stuss et al., 2006). Stephan et al. (2017) reported that the WCST is one of the most sensitive neuropsychological tests to detect changes resulting from alcohol abuse. In this study, individuals with BD completed fewer categories on the WCST compared to those without BD, and there was a significant association between the AUDIT-K total score and number of categories completed.

The number of categories completed refers to the number of sequences of 10 consecutive correct matches to the criterion sorting category and is the most common index used to assess cognitive control on the WCST along with perseverative errors (Stuss et al., 2006). Therefore, the present results indicate that individuals with BD have difficulties in cognitive control and these difficulties seem to be related to prefrontal dysfunction.

We administered TMT, COWA and Stroop Color-Word Test to evaluate components of executive functions, such as controlled attention and interference control, in addition to the WCST. We did not observe any significant differences between the BD and non-BD groups on the measures except the WCST. One possible explanation for the present results is that the deleterious effect of BD on the executive functions could be observed in a measure which is complicated and requires high-order cognitive functions such as the WCST (Faustino et al., 2021).

In addition, it is known that cognitive or behavioral changes emerge after alterations of the brain structure and function (Rubia et al., 2001). In other words, the emergence of behavioral deficits in the neuropsychological tasks require a somewhat long period of BD. For example, Lopez-Caneda et al. (2013) observed that young adults with BD and non-BD exhibited different EEG pattern, and the difference was more pronounced after 2 years of maintenance of BD. Furthermore, Gil-Hernandez and Garcia-Moreno (2016) observed better performance on some of the neuropsychological tests including the Stroop test in adolescents with BD than those without BD. The authors suggested that neurotoxic effects of BD on prefrontal cortex can be less evident in adolescence, but if BD persists the executive function would be exacerbated. As participants in the present study had a relatively short period of BD (the mean number of years of alcohol consumption in the BD group was 2.52), significant structural/functional alterations of brain and the resultant cognitive dysfunctions such as controlled attention or interference control could not be found.

The present study had several limitations that should be addressed in future studies. First, although the gender ratio between the BD and non-BD groups did not differ (χ^2^ = 0.874), we could not include equal numbers of male and female participants in each group. As significant differences in brain activation (Squeglia et al., 2011) and neuropsychological performance (Hartley et al., 2004; Townshend and Duka, 2005) between male and female binge drinkers have been observed, studies examining gender differences would provide insight into the nuanced effects of alcohol consumption on brain functions and cognition in male and female binge drinkers. Second, the important question of whether cognitive difficulties are present prior to BD and predict its onset, or whether BD induces the cognitive difficulties, has been posed recently. However, because of the cross-sectional and exploratory nature of this study, the direction of cognitive difficulties and BD onset cannot be ascertained. Future prospective longitudinal studies could provide answers to this question. Finally, college students who engage in BD are more likely to use other substances, including cigarettes and marijuana (Jones et al., 2001), so these substances should be controlled for in future studies.

In conclusion, college students with BD exhibited significantly poorer performance in the long-term free-recall condition of the K-CVLT and delayed recall condition of the RCFT, and completed fewer categories on the WCST than those without BD. In addition, there were significant negative associations of the AUDIT-K total score and AUQ binge score with the score in the long-term free-recall condition of the K-CVLT, between the scores for the ADUIT-K and delayed recall condition of the RCFT, and between the AUDIT-K score and number of completed categories on the WCST. These results indicate that college students with BD have difficulties with verbal/non-verbal memory and executive functions, which are controlled mainly by the hippocampus and prefrontal cortex, respectively. The present results also provide valuable information about the deleterious effects of BD on memory and executive function, even when the duration of BD is relatively short. Therefore, efforts reducing excessive alcohol consumption among college students should be done *via* individual interventions such as mobile technology-based interventions (Fowler et al., 2016) or structural interventions including college policies restricting places for alcohol consumption (Toomey et al., 2007).

## Data Availability Statement

The raw data supporting the conclusions of this article will be made available by the authors, without undue reservation.

## Ethics Statement

The study involving human participants was reviewed and approved by Sungshin Women’s University Institutional Review Board (SSWUIRB, 2019-020). The patients/participants provided their written informed consent to participate in this study.

## Author Contributions

M-SK helped in conceptualization, funding acquisition, writing of the manuscript, and supervised the overall aspects of the manuscript. J-GK contributed to recruit participants, administer, and score neuropsychological tests and interpretation of the results, and writing of the manuscript. Both authors contributed to the article and approved the submitted version.

## Conflict of Interest

The authors declare that the research was conducted in the absence of any commercial or financial relationships that could be construed as a potential conflict of interest.

## Publisher’s Note

All claims expressed in this article are solely those of the authors and do not necessarily represent those of their affiliated organizations, or those of the publisher, the editors and the reviewers. Any product that may be evaluated in this article, or claim that may be made by its manufacturer, is not guaranteed or endorsed by the publisher.
